# The functional role of long non-coding RNA in human carcinomas

**DOI:** 10.1186/1476-4598-10-38

**Published:** 2011-04-13

**Authors:** Ewan A Gibb, Carolyn J Brown, Wan L Lam

**Affiliations:** 1 British Columbia Cancer Agency Research Centre, Vancouver, Canada; 2 Department of Medical Genetics, University of British Columbia, Vancouver, Canada; 3 Department of Pathology and Laboratory Medicine, University of British Columbia, Vancouver, Canada

## Abstract

Long non-coding RNAs (lncRNAs) are emerging as new players in the cancer paradigm demonstrating potential roles in both oncogenic and tumor suppressive pathways. These novel genes are frequently aberrantly expressed in a variety of human cancers, however the biological functions of the vast majority remain unknown. Recently, evidence has begun to accumulate describing the molecular mechanisms by which these RNA species function, providing insight into the functional roles they may play in tumorigenesis. In this review, we highlight the emerging functional role of lncRNAs in human cancer.

## Introduction

One of modern biology's great surprises was the discovery that the human genome encodes only ~20,000 protein-coding genes, representing <2% of the total genome sequence [1,2]. However, with the advent of tiling resolution genomic microarrays and whole genome and transcriptome sequencing technologies it was determined that at least 90% of the genome is actively transcribed [3,4]. The human transcriptome was found to be more complex than a collection of protein-coding genes and their splice variants; showing extensive antisense, overlapping and non-coding RNA (ncRNA) expression [5-10]. Although initially argued to be spurious transcriptional noise, recent evidence suggests that the proverbial "dark matter" of the genome may play a major biological role in cellular development and metabolism [11-17]. One such player, the newly discovered long non-coding RNA (lncRNA) genes, demonstrate developmental and tissue specific expression patterns, and aberrant regulation in a variety of diseases, including cancer [18-27].

NcRNAs are loosely grouped into two major classes based on transcript size; small ncRNAs and lncRNAs (Table 1) [28-30]. Small ncRNAs are represented by a broad range of known and newly discovered RNA species, with many being associated with 5' or 3' regions of genes [4,31,32]. This class includes the well-documented miRNAs, RNAs ~22 nucleotides (nt) long involved in the specific regulation of both protein-coding, and putatively non-coding genes, by post-transcriptional silencing or infrequently by activation [33-35]. miRNAs serve as major regulators of gene expression and as intricate components of the cellular gene expression network [33-38]. Another newly described subclass are the transcription initiation RNAs (tiRNAs), which are the smallest functional RNAs at only 18 nt in length [39,40]. While a number of small ncRNAs classes, including miRNAs, have established roles in tumorigenesis, an intriguing association between the aberrant expression of ncRNA satellite repeats and cancer has been recently demonstrated [41-46].

**Table 1:** Types of human non-coding RNAs

Type	Subclasses	Symbol	References
	Transfer RNAs	tRNAs	[222]
	MicroRNAs	miRNAs	[33,34]
	Ribosomal 5S and 5.8S RNAs	rRNAs	[223,224]
	Piwi interacting RNAs	piRNAs	[38,225]
	Tiny transcription initiation RNAs	tiRNAs	[39,40]
	Small interfering RNAs	siRNA	[32]
	Promoter-associated short RNAs	PASRs	[144,149]
	Termini-associated short RNAs	TASRs	[144,149]
	Antisense termini associated short RNAs	aTASRs	[226]
Small ncRNA (18 to 200 nt in size)	Small nucleolar RNAs	snoRNAs	[227,228]
	Transcription start site antisense RNAs	TSSa-RNAs	[229]
	Small nuclear RNAs	snRNAs	[230]
	Retrotransposon-derived RNAs	RE-RNAs	[231,232]
	3'UTR-derived RNAs	uaRNAs	[145]
	x-ncRNA	x-ncRNA	[233]
	Human Y RNA	hY RNA	[234]
	Unusually small RNAs	usRNAs	[235]
	Small NF90-associated RNAs	snaRs	[236,237]
	Vault RNAs	vtRNAs	[238]

	Ribosomal 18S and 28S RNAs	rRNAs	[223,224]
	Long or large intergenic ncRNAs	lincRNAs	[8,58]
	Transcribed ultraconserved regions	T-UCRs	[85]
	Pseudogenes	none	[239,240]
	GAA-repeat containing RNAs	GRC-RNAs	[241]
Long ncRNA (lncRNAs, 200 nt to >100 kb in size)	Long intronic ncRNAs	none	[242,243]
	Antisense RNAs	aRNAs	[244]
	Promoter-associated long RNAs	PALRs	[144,149]
	Promoter upstream transcripts	PROMPTs	[245]
	Stable excised intron RNAs	none	[56]
	Long stress-induced non-coding transcripts	LSINCTs	[25]

In contrast to miRNAs, lncRNAs, the focus of this article, are mRNA-like transcripts ranging in length from 200 nt to ~100 kilobases (kb) lacking significant open reading frames. Many identified lncRNAs are transcribed by RNA polymerase II (RNA pol II) and are polyadenylated, but this is not a fast rule [47,48]. There are examples of lncRNAs, such as the antisense *asOct4-pg5 *or the brain-associated *BC200*, which are functional, but not polyadenylated [49-51]. Generally, lncRNA expression levels appear to be lower than protein-coding genes [52-55], and some lncRNAs are preferentially expressed in specific tissues [21]. However, recent findings have suggested novel lncRNAs may contribute a significant portion of the aforementioned 'dark matter' of the human transcriptome [56,57]. In an exciting report by Kapranov *et.al.*, it was revealed the bulk of the relative mass of RNA in a human cell, exclusive of the ribosomal and mitochondrial RNA, is represented by non-coding transcripts with no known function [57].

**Figure 1:**
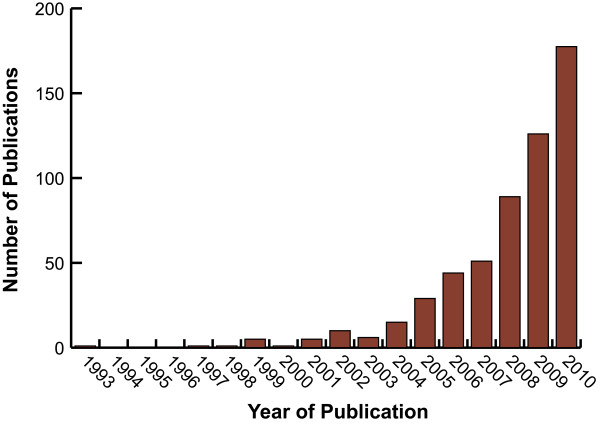
**Publications describing cancer-associated ncRNAs**. Entries are based on a National Library of Medicine Pubmed search using the terms "ncRNA" or "non-coding RNA" or "noncoding RNA" or non-protein-coding RNA" with cancer and annual (Jan.1-Dec.31) date limitations.

Like miRNAs and protein-coding genes, some transcriptionally active lncRNA genes display histone H3K4 trimethylation at their 5'-end and histone H3K36 trimethylation in the body of the gene [8,58,59]. The small number of characterized human lncRNAs have been associated with a spectrum of biological processes, for example, epigenetics, alternative splicing, nuclear import, as structural components, as precursors to small RNAs and even as regulators of mRNA decay [4,60-70]. Furthermore, accumulating reports of misregulated lncRNA expression across numerous cancer types suggest that aberrant lncRNA expression may be a major contributor to tumorigenesis [71]. This surge in publications reflects the increasing attention to this subject (Figure 1) and a number of useful lncRNA databases have been created (Table 2). In this review we highlight the emerging functional role of aberrant lncRNA expression, including transcribed ultraconserved regions (T-UCRs), within human carcinomas.

**Table 2:** Publically available long non-coding RNA online databases

Database Name	Website	Reference
ncRNAimprint	http://rnaqueen.sysu.edu.cn/ncRNAimprint/	[246]
ncRNAdb	http://research.imb.uq.edu.au/rnadb/	[247]
Functional RNAdb	http://www.ncrna.org/frnadb/	[248]
NONCODE	http://www.noncode.org/	[249]
lncRNA db	http://longnoncodingrna.com/	[250]
Rfam	http://rfam.sanger.ac.uk/	[251]
NRED	http://jsm-research.imb.uq.edu.au/nred/cgi-bin/ncrnadb.pl	[252]
Ncode (Invitrogen)	http://escience.invitrogen.com/ncRNA/	N/A
NcRNA Database	http://biobases.ibch.poznan.pl/ncRNA/	[253]
T-UCRs	http://users.soe.ucsc.edu/~jill/ultra.html	[202]
NPInter	http://www.bioinfo.org.cn/NPInter/	[254]

## Background on Long Non-Coding RNA

### Nomenclature and Classification of LncRNA

The definition 'non-coding RNA' is typically used to describe transcripts where sequence analysis has failed to identify an open reading frame. However, one should exercise caution when exploring putative non-coding transcripts, as there are cases where 'non-coding' transcripts were found to encode short, functional peptides [72]. Currently, a universal classification scheme to define lncRNAs does not exist and therefore there are numerous synonyms describing the same type of transcripts. Terms such as large non-coding RNA, mRNA-like long RNA, and intergenic RNA all define cellular RNAs, exclusive of rRNAs, greater than 200 nt in length and having no obvious protein-coding capacity [62]. Consequently, this has led to confusion in the literature as to exactly which transcripts should constitute a lncRNA. For example, one subclass of lncRNAs is called large or long intergenic ncRNAs (lincRNAs). These lncRNAs are exclusively intergenic and are marked by a chromatin signature indicative of transcription [8,58]. To further compound classification confusion, RNA species that are bifunctional preclude categorization into either group of protein-coding or ncRNAs as their transcripts function both at the RNA and protein levels [73]. In these rare cases, they are classified on a case-by-case basis, reserving the term 'lncRNA' to describe transcripts with no protein-coding capacity. In the meantime, and for the purposes of this review, we will consider lncRNAs as a blanket term to encompass mRNA-like ncRNAs, lincRNAs, as well as antisense and intron-encoded transcripts, T-UCRs and transcribed pseudogenes.

### Discovery of LncRNAs

**Figure 2:**
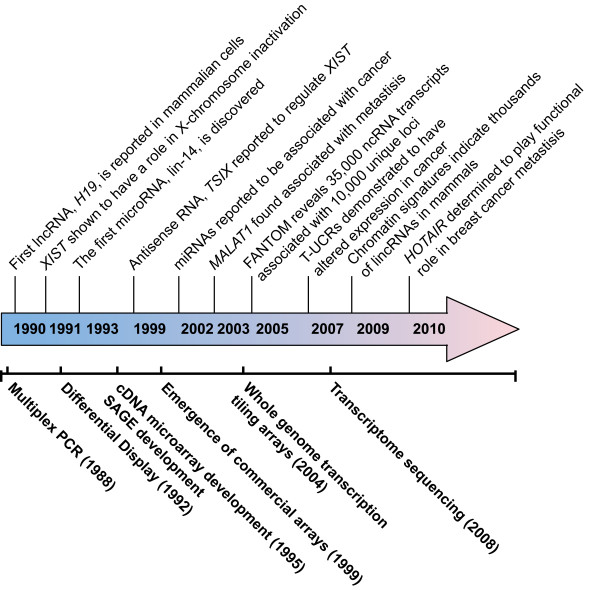
**Timeline of cancer-associated ncRNA discoveries relative to transcriptome analysis technologies (not drawn to scale)**.

The earliest reports describing lncRNA predated the discovery of miRNAs, although the term 'lncRNA' had not been coined at the time (Figure 2). One of the first lncRNA genes reported was the imprinted *H19 *gene, which was quickly followed by the discovery of the silencing X-inactive-specific transcript *(XIST) *lncRNA gene, which plays a critical function in X-chromosome inactivation [74,75]. However, the discovery of the first miRNA *lin-14 *dramatically redirected the focus of ncRNA research from long ncRNAs to miRNAs [76]. Moreover, during this time the discovery of miRNAs revealed RNA could regulate gene expression and later that entire gene networks could be affected by ncRNA expression and within the last decade miRNAs were discovered to be associated with cancer (Figure 2) [76-79]. At the time of this writing there are approximately 1049 human miRNAs described in miRBase V16 [80,81] with the potential of affecting the expression of approximately 60% of protein -coding genes [82,83]. Conversely, the variety and dynamics of lncRNA expression was not to be fully appreciated until the introduction of whole transcriptome sequencing. With the advent of the FANTOM and ENCODE transcript mapping projects, it was revealed that the mammalian genome is extensively transcribed, although a large portion of this represented non-coding sequences [3,84]. Coupled with the novel functional annotation of a few lncRNAs, this discovery promoted research focusing on lncRNA discovery and characterization. Recent reports have described new lncRNA classes such as lincRNAs and T-UCRs [8,58,85]. Current estimates of the lncRNA gene content in the human genome ranges from ~7000 - 23,000 unique lncRNAs, implying this class of ncRNA will represent an enormous, yet undiscovered, component of normal cellular networks that may be disrupted in cancer biology [62].

### Emerging Role of Long Non-Coding RNA in Tumorigenesis

A role for differential lncRNA expression in cancer had been suspected for many years, however, lacked strong supporting evidence [86]. With advancements in cancer transcriptome profiling and accumulating evidence supporting lncRNA function, a number of differentially expressed lncRNAs have been associated with cancer (Table 3). LncRNAs have been implicated to regulate a range of biological functions and the disruption of some of these functions, such as genomic imprinting and transcriptional regulation, plays a critical role in cancer development. Here we describe some of the better characterized lncRNAs that have been associated with cancer biology.

**Table 3:** Human cancer-associated lncRNAs

LncRNA	Size	Cytoband	Cancer types	References
*HOTAIR*	2158 nt	12q13.13	Breast	[18,68]
*MALAT1*/α/*NEAT2*	7.5 kb	11q13.1	Breast, lung, uterus, pancreas, colon, prostate, liver, cervix^1^, neuroblastoma^1^, osteosarcoma	[135,137-139,152,255,256]
*HULC*	500 nt	6p24.3	Liver, hepatic colorectal metastasis	[170,171]
*BC200*	200 nt	2p21	Breast, cervix, esophagus, lung, ovary, parotid, tongue	[50,51]
*H19*	2.3 kb	11p15.5	Bladder, lung, liver, breast, endometrial, cervix, esophagus, ovary, prostate, choricarcinoma, colorectal	[74,92,95,97,102,103,257-264]
*BIC/MIRHG155/MIRHG2*	1.6 kb	21q11.2	B-cell lymphoma	[153]
*PRNCR1*	13 kb	8q24.2	Prostate	[187]
*LOC285194*	2105 nt	3q13.31	Osteosarcoma	[265]
*PCGEM1*	1643 nt	2q32.2	Prostate	[188,266,267]
*UCA1*/*CUDR*	1.4 kb, 2.2 kb, 2.7 kb	19p13.12	Bladder, colon, cervix, lung, thyroid, liver, breast, esophagus, stomach	[268-270]
*DD3*/*PCA3*	0.6 kb, 2 kb, 4 kb	9q21.22	Prostate	[189,190]
*anti-NOS2A*	~1.9 kb	17q23.2	Brain^1^	[271]
*uc.73A*	201 nt	2q22.3	Colon	[200]
*TUC338 *(encodes uc.338)	590 nt	12q13.13	Liver	[203]
*ANRIL*/*p15AS*/*CDK2BAS*	34.8 kb & splice variants	9p21.3	Prostate, leukemia	[175,176,183,272]
*MEG3*	1.6 kb & splicing isoforms	14q32.2	Brain (downregulated)	[156-158,162]
*GAS5*/*SNHG2*	Multiple isoforms	1q25.1	Breast (downregulated)	[273]
*SRA-1*/*SRA *(bifunctional)	1965 nt	5q31.3	Breast, uterus, ovary (hormone responsive tissue)	[274,275]
*PTENP1*	~3.9 kb	9p13.3	Prostate	[173,174]
*ncRAN*	2186 nt, 2087 nt	17q25.1	Bladder, neuroblastoma	[276,277]

^1 ^Cell lines

#### Imprinted lncRNA genes

Imprinting is a process whereby the copy of a gene inherited from one parent is epigenetically silenced [87,88]. Intriguingly, imprinted regions often include multiple maternal and paternally expressed genes with a high frequency of ncRNA genes. The imprinted ncRNA genes are implicated in the imprinting of the region by a variety of mechanisms including enhancer competition and chromatin remodeling [89]. A key feature of cancer is the loss of this imprinting resulting in altered gene expression [90,91]. Two of the best known imprinted genes are in fact lncRNAs.

##### H19

The *H19 *gene encodes a 2.3 kb lncRNA that is expressed exclusively from the maternal allele. *H19 *and its reciprocally imprinted protein-coding neighbor the Insulin-Like Growth Factor 2 or *IGF2 *gene at 11p15.5 were among the first genes, non-coding or otherwise, found to demonstrate genomic imprinting [74,92]. The expression of *H19 *is high during vertebrate embryo development, but is downregulated in most tissues shortly after birth with the exception of skeletal tissue and cartilage [20,93,94]. Loss of imprinting and subsequent strong gene expression has been well-documented in human cancers. Likewise, loss of imprinting at the *H19 *locus resulted in high *H19 *expression in cancers of the esophagus, colon, liver, bladder and with hepatic metastases [95-97].

*H19 *has been implicated as having both oncogenic and tumor suppression properties in cancer. *H19 *is upregulated in a number of human cancers, including hepatocellular, bladder and breast carcinomas, suggesting an oncogenic function for this lncRNA [97-99]. In colon cancer *H19 *was shown to be directly activated by the oncogenic transcription factor c-Myc, suggesting *H19 *may be an intermediate functionary between c-Myc and downstream gene expression [98]. Conversely, the tumor suppressor gene and transcriptional activator p53 has been shown to down-regulate *H19 *expression [100,101]. *H19 *transcripts also serve as a precursor for miR-675, a miRNA involved in the regulation of developmental genes [102]. miR-675 is processed from the first exon of *H19 *and functionally downregulates the tumor suppressor gene retinoblastoma (*RB1*) in human colorectal cancer, further implying an oncogenic role for *H19 *[103].

There is evidence suggesting *H19 *may also play a role in tumor suppression [104,105]. Using a mouse model for colorectal cancer, it was shown that mice lacking *H19 *manifested an increased polyp count compared to wild-type [106]. Secondly, a mouse teratocarcinoma model demonstrated larger tumor growth when the embryo lacked *H19*, and finally in a hepatocarcinoma model, mice developed cancer much earlier when *H19 *was absent [107]. The discrepancy as to whether *H19 *has oncogenic or tumor suppressive potential may be due in part to the bifunctional nature of the lncRNA or may be context dependent. In either case, the precise functional and biological role of *H19 *remains to be determined.

##### XIST - X-inactive-specific transcript

The 17 kb lncRNA *XIST *is arguably an archetype for the study of functional lncRNAs in mammalian cells, having been studied for nearly two decades. In female cells, the *XIST *transcript plays a critical role in X-chromosome inactivation by physically coating one of the two X-chromosomes, and is necessary for the cis-inactivation of the over one thousand X-linked genes [75,108-110]. Like the lncRNAs *HOTAIR *and *ANRIL, XIST *associates with polycomb-repressor proteins, suggesting a common pathway of inducing silencing utilized by diverse lncRNAs. In mice, X inactivation in the extraembryonic tissues is non-random, and the initial expression of *Xist *is always paternal in origin, followed later by random X inactivation in the epiblast associated with random mono-allelic expression [111]. Intriguingly, regulation of *Xist *in mouse has been shown to be controlled by interactions amongst additional ncRNAs, including an antisense transcript, *Tsix*, enhancer-associated ncRNAs (*Xite*) and the upstream *Jpx *and *Ftx *lncRNAs [109,112-115]. Intriguingly, murine *Xist *and *Tsix *duplexes are processed into small RNAs by an apparently Dicer-dependent manner, suggesting a significant overlap between the regulatory networks of both lncRNAs and sRNAs [116]. It is unclear, however, how much of this regulation is conserved in humans, who do not show imprinted X inactivation [117]. While *XIST *expression levels are correlated with outcome in some cancers, such as the therapeutic response in ovarian cancer [118], the actual role that *XIST *may play in human carcinomas, if any, is not entirely clear.

There is generally believed to be only a limited developmental window in which X inactivation can occur, and loss of *XIST *from an inactive X chromosome does not result in reactivation of the X chromosome [119,120]. Thus, tumors with additional X chromosomes generally keep the inactivation status of the duplicated X. For tumors in which two active X chromosomes are observed, as has been frequently observed in breast cancer, the most common mechanism involves loss of the inactive X and duplication of the active X, often resulting in heterogeneous *XIST *expression in these tumors [121-123]. Loss of the inactive X and abnormalities of *XIST *expression may be indicative of more general heterochromatin defects [124]. Analogously, failure to properly reset *XIST *and X inactivation may serve as a marker of proper resetting of epigenetic marks in stem cells or induced pluripotent stem cells [125,126]. Correlations between XIST expression and cancer state can also occur spuriously. Notably, in cytogenetically normal cells, *XIST *is found only in females as males do not have an inactive X. Therefore in cancers correlations can be observed with *XIST *expression due to differential presence of male or female samples, and many cancers do show different onsets and progressions in males and females. Furthermore, *XIST *expression will increase with the number of inactive X chromosomes. While it might be anticipated that there would be little advantage to a tumor acquiring inactive chromosomes, it has been shown that approximately 15% of human X-linked genes continue to be expressed from the inactive X chromosome [127].

#### Involvement in metastasis

##### HOTAIR - HOX antisense intergenic RNA

Several lncRNAs have been implicated in metastasis. One of the first lncRNAs described to have a fundamental role in cancer was the metastasis-associated *HOX Antisense Intergenic RNA *(*HOTAIR*), a 2.2 kb gene located in the mammalian *HOXC *locus on chromosome 12q13.13 [18]. This lncRNA was found to be highly upregulated in both primary and metastatic breast tumors, demonstrating up to 2000-fold increased transcription over normal breast tissue [68]. High levels of *HOTAIR *expression were found to be correlated with both metastasis and poor survival rate, linking a ncRNA with cancer invasiveness and patient prognosis [68]. Furthermore, it was demonstrated that if cells expressing *HOTAIR *were grafted into mouse mammary fat pads, a modest increase in the rate of primary tumor growth was observed [68]. Interestingly, there are reports indicating that numerous lncRNAs are transcribed from the *HOX *locus, suggesting that *HOTAIR *may be only one example of a global regulatory phenomena [58].

**Figure 3:**
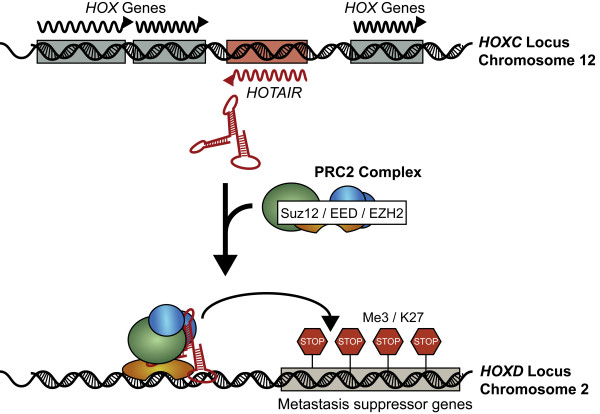
**Proposed mechanism of *HOTAIR *mediated gene silencing of 40 kb of the *HOXD *locus, which is involved in developmental patterning**. The *HOTAIR *lncRNA is transcribed from the *HOXC *locus and functions in the binding and recruitment and binding of the PRC2 and LSD1 complex to the *HOXD *locus. For clarity, only the PRC2 complex is indicated in the above figure. Through an undetermined mechanism, the *HOTAIR*-PRC2-LSD1 complex is redirected to the *HOXD *locus on chromosome 2 where genes involved in metastasis suppression are silenced through H3K27 methylation and H3K4 demethylation. This drives breast cancer cells to develop gene expression patterns that more closely resemble embryonic fibroblasts than epithelial cells.

The spliced and polyadenylated *HOTAIR *RNA does not encode any proteins but has been demonstrated to be intimately associated with the mammalian polycomb repressive complex 2 (PRC2) which is comprised of the H3K27 methylase EZH2, SUZ12 and EED [68,128,129] (Figure 3). Polycomb group proteins mediate repression of transcription of thousands of genes controlling differentiation pathways during development, and have roles in stem cell pluripotency and human cancer [68,130-133].

*HOTAIR *binding results in a genome-wide re-targeting of the PRC2 complex. The *HOXD *locus on chromosome 2 is a PRC2 target (Figure 3). The consequence of PRC2/*HOTAIR *localization is the transcriptional silencing of a 40 kb region of the *HOXD *locus which remodels the gene expression pattern of breast epithelial cells to more closely resemble that of embryonic fibroblasts [68]. The *HOTAIR *RNA appears to act as a molecular scaffold, binding at least two distinct histone modification complexes. The 5' region of the RNA binds the PRC2 complex responsible for H3K27 methylation, while the 3' region of *HOTAIR *binds LSD1, a histone lysine demethylase that mediates enzymatic demethylation of H3K4Me2 [128,134]. Although, the precise mechanism of *HOTAIR *activities remains to be elucidated, it is clear that *HOTAIR *reprograms chromatin state to promote cancer metastasis.

##### MALAT1 - Metastasis-associated lung adenocarcinoma transcript 1

The *MALAT1 *gene, or *metastasis-associated lung adenocarcinoma transcript 1*, was first associated with high metastatic potential and poor patient prognosis during a comparative screen of non-small cell lung cancer patients with and without metastatic tumors [135]. This lncRNA is widely expressed in normal human tissues [135,136] and is found to be upregulated in a variety of human cancers of the breast, prostate, colon, liver and uterus [27,137-139]. Notably, the *MALAT1 *locus at 11q13.1 has been identified to harbor chromosomal translocation breakpoints associated with cancer [140-142].

**Figure 4:**
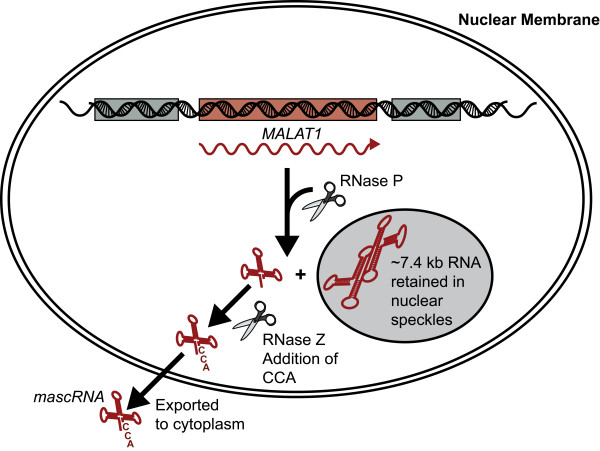
**Expression and processing of *MALAT1 *transcripts**. Full length 7.5 kb *MALAT1 *RNA is processed by RNaseP and RNaseZ to generate the small ncRNA *mascRNA*, which is then exported to the cytoplasm. The larger *MALAT1 *RNA is retained in the nuclear speckles where it is thought to have a role in regulating alternative splicing machinery.

Intriguingly, cellular *MALAT1 *transcripts are subject to post-transcriptional processing to yield a short, tRNA-like molecule *mascRNA *and a long *MALAT1 *transcript with a poly(A) tail-like moiety [143] (Figure 4). Using the mouse homologue to the human *MALAT1 *gene, it was revealed that ribonuclease (RNase) P processing generates the 3' end of the long *MALAT1 *transcript and the 5' end of the *mascRNA*. The shorter *mascRNA *adopts a tRNA clover-leaf structure and is subject to RNaseZ processing and the addition of a CCA to its 3' end before being exported to the cytoplasm. The generation of putatively functional sRNAs by post-transcriptional processing of lncRNAs, such *mascRNA *from *MALAT1*, may be reflective of a central, unexplored theme in ncRNA biology [144]. Recent evidence suggests regulated post-transcriptional processing events, such as splicing and post-cleavage capping, may increase the functional diversity of the metazoan transcriptome [145-149]. In contrast, the long *MALAT1 *transcript is not polyadenylated, but has a poly(A) tail-like sequence that is genome encoded and is putatively present to protect the *MALAT1 *transcript from degradation. Moreover, *MALAT1 *localizes to nuclear speckles in a transcription dependent manner [136,150].

Following the correlation between high levels of *MALAT1 *expression and metastasis, a number of studies have implicated *MALAT1 *in the regulation of cell mobility. For example, RNA interference (RNAi)-mediated silencing of *MALAT1 *impaired the *in vitro *migration of lung adenocarcinoma cells through concomitant regulation of motility regulated genes via transcriptional and/or post-transcriptional means [151]. Similarly, short hairpin RNA inhibition of *MALAT1 *reduced cell proliferation and invasive potential of a cervical cancer cell line [152]. Collectively, these studies suggest that *MALAT1 *regulates the invasive potential of metastatic tumor cells. Other examples of cancer-associated lncRNAs subject to post-transcriptional processing include the miR-155 host gene *BIC *and the miR-17-92 cluster in B-cell lymphoma and neuroblastoma, respectively [153,154].

Recent efforts have focused on elucidating the molecular role of nuclear speckle localized *MALAT1 *transcripts, while the function for the *mascRNA *has yet to be determined. Nuclear speckles are thought to be involved in the assembly, modification and/or storage of pre-mRNA processing machinery [136,155]. A recent publication revealed *MALAT1 *RNA strongly associates with serine-arginine rich splicing factor (SR) proteins which are involved in both constitutive and alternative splicing and the levels of *MALAT1 *regulated the cellular levels of phosphorylated SR proteins [66]. These findings imply that the lncRNA *MALAT1 *may serve a function in the regulation of alternate splicing by modulating the activity of SR proteins, although precisely how this may contribute to tumorigenesis remains unknown.

#### LncRNA-mediated tumor suppression by P53 stimulation

##### MEG3 - Maternally expressed gene 3

The *maternally expressed gene 3 *(*MEG3*) was the first lncRNA proposed to function as a tumor suppressor. The *MEG3 *gene is expressed in many normal human tissues, with the highest expression in the brain and pituitary gland [156,157]. *MEG3 *expression was not detectable in various brain cancers, nor in a range of human cancer cell lines implicating a potential role of this lncRNA in suppression of cell growth. Moreover, ectopic expression of *MEG3 *RNA was found to suppress the growth of several human cancer cell lines, further supporting the role of *MEG3 *as a tumor suppressor [157]. In clinically nonfunctioning pituitary tumors, it was demonstrated that hypermethylation of the *MEG3 *regulatory region was associated with the loss of *MEG3 *expression, providing evidence for a mechanism of *MEG3 *inactivation [157].

*MEG3 *is a paternally imprinted, single copy gene comprised of 10 exons [156]. To date, 12 *MEG3 *isoforms have been detected (*MEG3*; a-3k) due to alternative splicing [158]. Each isoform contains the common exons 1-3 and 8-10, but varies in the combination of exons 4-7 in the middle of the transcript [158]. Notably, the last intron of *MEG3 *encodes the evolutionarily conserved miR-770, a miRNA with a number of putative mRNA targets [159]. This arrangement mirrors the presence of miR-675 in *H19*, except in that instance the miRNA was exon encoded [102]. The originally identified isoform of *MEG3 *is expressed as a 1.6 kb polyadenylated transcript that is localized to the nucleus where it is associated with chromatin although some cytoplasmic *MEG3 *transcripts have been detected [156,160,161]. All 12 *MEG3 *isoforms demonstrate three distinct secondary folding motifs designated M1, M2 and M3 [158].

Functionally, *MEG3 *has been implicated as a top-level regulatory RNA due to its ability to stimulate both p53-dependent and p53-independent pathways [158,162]. Critically, the *MEG3*-mediated functional activation of p53 is dependent on the secondary structure of the *MEG3 *RNA rather than on primary sequence conservation. In an elegant series of experiments, Zhang *et al. *demonstrated that replacing particular regions of the *MEG3 *with unrelated sequence had no effect on the activation of p53 provided the original secondary structure was preserved [158]. This observation strengthens the argument that the rapid evolution and relative lack of sequence conservation does not limit the potential functionality of these unique RNA species [163]. Conversely, the novel lincRNA-p21 has been described as a downstream repressor in the p53 transcriptional response, suggesting the complex p53 transcriptional network includes numerous regulatory lncRNAs [164].

#### Depletion of miRNAs by a lncRNA 'molecular decoy' or 'miRNA sponge'

Expression of miRNAs in cancer can be deregulated by a range of mechanisms, including copy number alterations and epigenetic silencing [165-168]. Two recent examples have demonstrated that lncRNAs can act as natural 'miRNA sponges' to reduce miRNA levels [169].

##### HULC - Highly Upregulated in Liver Cancer

The most highly upregulated transcript found in a microarray-based study of gene expression in hepatocellular carcinoma was determined to be the ncRNA *HULC*, or *Highly Upregulated in Liver Cancer *[170]. Transcribed from chromosome 6p24.3, this lncRNA demonstrates the hallmarks of a typical mRNA molecule, including a single spliced GT-AG intron, canonical polyadenylation signals upstream of the poly(A) tail and nuclear export demonstrating strong localization to the cytoplasm. Although *HULC *was found to co-purify with ribosomes, no translation product for this lncRNA has been detected, supporting its classification as a non-coding transcript [170]. In addition to liver cancer, *HULC *was found to be highly upregulated in hepatic colorectal cancer metastasis and in hepatocellular carcinoma cell lines (HCC) producing hepatitis B virus (HBV) [171].

**Figure 5:**
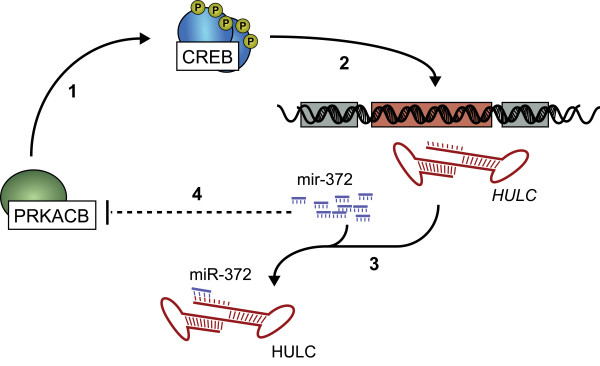
**Proposed mechanism of *HULC *upregulation in hepatocellular carcinoma**. (1) The kinase PRKACB functions as an activator of CREB. (2) Phosphorylated (activated) CREB forms part of the RNA pol II transcriptional machinery to activate *HULC *expression. (3) Abundant *HULC *RNA acts as a molecular sponge to sequester and inactivate the repressive function miR-372. (4) PRKACB levels increase, as transcripts are normally translationally repressed by high miR-372 levels.

A recent paper began to elucidate the mechanism of upregulation of *HULC *in liver cancer cells, and to provide a potential mechanism of *HULC *function [172]. *HULC *exists as part of an intricate auto-regulatory network, which when perturbed, resulted in increased *HULC *expression (Figure 5). The *HULC *RNA appeared to function as a 'molecular decoy' or 'miRNA sponge' sequestering miR-372, of which one function is the translational repression of PRKACB, a kinase targeting cAMP response element binding protein (CREB). Once activated, the CREB protein was able to promote *HULC *transcription by maintaining an open chromatin structure at the *HULC *promoter resulting in increased *HULC *transcription [172].

##### Pseudogene pairs: PTEN and PTENP1

The discovery that *HULC *can act as a molecular decoy or 'miRNA sponge' mirrors a recent report describing an intricate relationship between the tumor suppressor phosphatase and tensin homolog *(PTEN) *and the matching lncRNA *phosphatase and tensin homolog pseudogene 1 (PTENP1) *[173]. The RNA transcripts of the *PTEN*/*PTENP1 *pair share similar 3' untranslated regions (3'UTRs) which both bind the same miRNAs. By binding miRNAs, *PTENP1 *transcripts reduce the effects of translational repression on PTEN therefore allowing expression of this tumor suppressor. In cancer, specific mutations inactivate these miRNA binding sites in *PTENP1*, therefore reducing the translation of *PTEN *and promoting tumor growth [173]. This is especially pertinent as subtle changes in *PTEN *levels can influence cancer susceptibility [174].

#### LncRNA-mediated deregulation of the tumor suppressors

##### ANRIL - Antisense Non-coding RNA in the INK4 Locus

Located as part of the 42kb *INK4b-ARF-INK4a *locus on chromosome 9p21.3, the *Antisense Non-coding RNA in the INK4 Locus *(*ANRIL*) is transcribed by RNA pol II and processed into alternatively spliced isoforms, including an unspliced transcript of 34.8 kb termed p15AS [175,176]. The *INK4b-ARF-INKa *locus has an important role in cell cycle control, cell senescence, stem cell renewal and apoptosis through P14ARF-MDM2-P53 and P16*Ink4a*/p15*Ink4b*-Cdk4/6-pRb pathways [177-179]. A mouse model suggests that the well-characterized tumor suppressor genes encoded within this locus are regulated by Polycomb proteins [180].

Aberrant expression and single nucleotide polymorphisms (SNPs) within *ANRIL *have been associated with susceptibility to a range of human diseases, including cancer [181,182]. Moreover, the *INK4b-ARF-INK4a *locus is subject to frequent deletion or hypermethylation in cancers, including leukemia, melanoma, lung and bladder cancers [181]. *ANRIL *has been associated with epigenetic silencing of the tumor suppressor gene *p15*, although the molecular events leading to this silencing were unclear [175].

Evidence has suggested an intriguing mechanisms for *ANRIL*-mediated silencing of the *INK4b-ARF-INK4a *locus. Recently an interaction between chromatin modifying PRC complexes and *ANRIL *has been described, further elucidating the regulatory mechanism of this lncRNA. Like the lncRNA *HOTAIR *which binds both the polycomb repressor complex PRC2 and the LSD1 complex, *ANRIL *binds and recruits two polycomb repressor complexes modifying complexes, PRC1 and PRC2 [183,184]. This resulted in ANRIL/PRC mediated silencing of the genes in the *INK4b-ARF-INK4a *locus. While the distinct regions of the *HOTAIR *lncRNA required for interactions with each protein complex were determined [128], future studies will be necessary to elucidate the structural requirements of lncRNAs such as *ANRIL *with chromatin regulators such as PRC1/PRC2.

The molecular details of *ANRIL*-mediated tumor suppression are becoming more clear. However, other mechanisms of lncRNA-mediated suppression of tumor suppressor genes have been reported. For example, a change in the expression ratio of bidirectional genes has been shown to mediate the expression of the tumor suppressor p21 [185]. Collectively, these observations suggest that lncRNA-mediated silencing of tumor suppressor genes may be a major mechanism driving tumorigenesis.

#### Cancer type specific lncRNA expression

Many of the described lncRNAs are expressed in a variety of cancers, however a select few thus far have been associated with a single cancer type. *HOTAIR*, for example, has only been described in breast cancer, while three lncRNAs *PCGEM1, DD3 *and *PCNCR1 *have been associated solely with prostate cancer [68,186,187]. The most recently described of these, *Prostate Cancer Non-Coding RNA 1 *(*PCNCR1*) lncRNA, was identified in a 'gene desert' on chromosome 8q24.2 and is associated with susceptibility to prostate cancer. *PCNCR1 *is expressed as an intronless, ~13 kb transcript with a potential role in trans-activation of androgen receptor (AR), a key player in prostate cancer progression [187]. Likewise, *PCGEM1 *(*Prostate Specific Gene 1*) was found to have properties supporting tumorigenesis, as ectopic overexpression of *PCGEM1 *RNA resulted in increased cell growth and colony formation in cell lines [188]. The lncRNA *Differential Display Code 3 *(*DD3*) is also highly over expressed in prostate cancer, yet little is known about the role *DD3 *may play in prostate cancer progression [189,190]. Finally, the liver associated lncRNA *HULC *is highly expressed in primary liver tumors, and in colorectal carcinomas that metastasized to the liver, but not in the primary colon tumors or in non-liver metastases [171].

#### RNA polymerase III transcription of lncRNA

The lncRNAs described thus far are products of RNA pol II transcription, yet many ncRNAs are transcribed by RNA polymerase III (RNA pol III) [191]. Importantly, RNA pol III is frequently deregulated in cancer cells resulting in increased activity [192,193]. The molecular mechanisms driving increased RNA pol III activity in tumor cells include overexpression of RNA pol III transcription factors, escape from RNA pol III repressors and direct oncogene-mediated activation [193-195]. Aberrant RNA pol III function may have consequences to the expression of lncRNAs transcribed by this polymerase.

For example, the lncRNA *BC200 *is a small cytoplasmic lncRNA in the neurons of primate nervous systems and human cancers, but not in non-neuronal organs [20,50,51,196,197]. Unlike the majority of lncRNAs described thus far, *BC200 *is transcribed by RNA pol III and shares unique homology with human Alu elements [196,198]. Similarly, the lncRNA *HULC *also shares homology with mobile DNA, in this case with a long terminal repeat (LTR) retroelement [170]. The *BC200 *RNA has been characterized as a negative regulator of eIF4A-dependent translation initiation [199].

Due to the fact that many whole transcriptome sequencing methods were developed to enrich for poly(A) purified transcripts, RNA pol III transcripts may have been excluded from analysis. This suggests that other, yet unidentified RNA pol III lncRNAs over-expressed in cancer may be participating in tumorigenesis.

#### Aberrant T-UCR expression in human carcinoma

**Figure 6:**
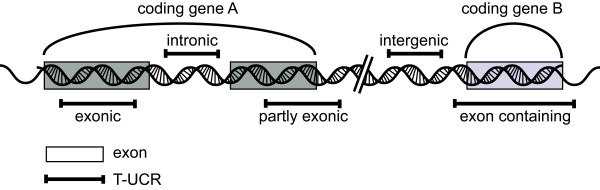
**Genomic locations of the five classes of T-UCRs**. The exons of coding genes are indicated by boxes, while the locations of the T-UCR elements are marked by a double-T bar. The five possible positions are as indicated exonic, partly exonic, exon containing, intronic and intergenic.

Transcribed ultraconserved Regions (T-UCRs) are evolutionary conserved sequences found in both intergenic and intragenic regions of the human genome [200,201]. These unique sequences are defined as 481 segments of DNA that are absolutely conserved between orthologous regions of the human, rat and mouse genomes [201,202]. The transcription products of T-UCRs are 200-779 nt in length and were originally classed into three categories, non-exonic, exonic and possibly exonic, according to their overlap with known protein-coding genes [202]. More recently, T-UCRs have been re-annotated into a more descriptive set of five categories: intergenic (38.7%), intronic (42.6%), exonic (4.2%), partly exonic (5%) or exonic containing (5.6%) (Figure 6) [59].

The high degree of conservation of T-UCRs, combined with their tissue-specific expression, suggests these ncRNAs may play a critical role in cellular metabolism and development [200]. The expression of many T-UCRs is significantly altered in cancer, notably in adult chronic lymphocytic leukemias, colorectal and hepatocellular carcinomas and neuroblastomas [85,200]. Their aberrant transcription profiles can be used to differentiate types of human cancers and have been linked to patient outcome [85]. Some of the T-UCRs reside in genomic regions associated with specific types of cancer [200]. For example, in colon cancer, the T-UCR *uc.73A *is one of the most highly upregulated T-UCRs, and this lncRNA has been found to show oncogenic properties by proliferation assays [200]. Similarly, T-UCR *uc.338 *is significantly upregulated in human hepatocarcinoma tumor and cell lines, and *uc.338 *was found to be part of a larger transcriptional unit coined *TUC338 *which is involved in cell growth [203].

The mechanism by which T-UCRs are differentially expressed is unclear. A study profiling T-UCRs in neuroblastomas did not find a consistent association between genomic alterations and T-UCR expression, suggesting aberrant T-UCR expression may be a consequence of epigenetic mechanisms. Like miRNAs and coding genes, T-UCR expression has been shown to be repressed by CpG island hypermethylation [85,204]. Similarly, miRNAs have been shown to bind to, and downregulate T-UCRs, suggesting a complex regulatory mechanism may exist between ncRNAs in human cancers [85,200]. Collectively, these reports have suggested that aberrant T-UCR expression may have a yet undetermined biological role in tumorigenesis.

### Utility of LncRNAs in Cancer Diagnostics and Therapies

Like their smaller non-coding miRNA counterparts, lncRNAs represent a significant untapped resource in terms of developing diagnostics and therapies. Differential or high level expression of certain cancer type-specific lncRNAs can be exploited for the development of novel biomarkers as lncRNA expression or may potentially correlate with patient response to chemotherapy. Understanding the mechanism(s) by which lncRNAs act will continue to provide novel approaches to regulating genes including the development of mimetics to compete with binding sites for miRNAs, chromatin remodelers, or DNA. It has been suggested that mediating transcriptional gene silencing (TGS) pathways, especially those of tumor suppressors or oncogenes, could be of high therapeutic benefit [205].

It has been widely reported that cancer-specific miRNAs are detectable in the blood, sputum and urine of cancer patients [206-210]. Likewise, lncRNAs have demonstrated utility as fluid-based markers of specific cancers. For example, the prostate specific lncRNA *DD3 *has been developed into highly specific, nucleic acid amplification-based marker of prostate cancer, which demonstrated higher specificity than serum prostate-specific antigen (PSA) [211,212]. Similarly, the highly expressed hepatocarcinoma-associated lncRNA *HULC *is detectable in the blood of hepatocarcinoma patients by conventional PCR methods [170].

The use of lncRNAs as therapeutic agents is only beginning to be explored [213]. Although our understanding of the molecular mechanisms of lncRNA function is limited, some features of lncRNAs make them ideal candidates for therapeutic intervention. Many lncRNAs appear to have protein-binding or functional potential that is dependent on secondary structure, this may provide a means of intervention [214]. Preventing the interactions of *HOTAIR *with the PRC2 or LSD1 complexes, for example, may limit the metastatic potential of breast cancer cells [215].

The tumor expression of certain lncRNAs provides a source of regulatory regions that can be used to reduce the risk of affecting normal tissues during transgene-mediated treatment. For example, *H19 *is strongly expressed in embryonic cells and in a wide-range of human cancers [20,93,94]. As such, a plasmid-based system has been developed to exploit the tumor-specific expression of *H19*, primarily tested in treating bladder cancer. A plasmid construct, harboring a diptheria toxin gene driven by *H19 *specific regulatory sequences, is either administered via intratumoral injection as naked DNA, or complexed to the cationic polymer polyethylenimine (PEI) to form a polyplex vector [216]. PEI-complexed plasmid is thought to increase the efficiency of DNA uptake via clathrin-dependent and -independent (cholesterol-dependent) pathways [217]. Upon uptake, high levels of diptheria toxin are expressed in the tumor, resulting in a reduction in tumor size in human trials [218-221]. Collectively, these advances indicate the potential in developing lncRNA mediated diagnostics and therapies.

## Conclusions

Differential expression of lncRNAs is becoming recognized as a hallmark feature in cancer, however the functional role for the vast majority of these unique genes is still in question. In this review, we highlight characterized lncRNAs described to play a functional role in cancer-associated processes, such as metastasis and loss of imprinting. Aberrant lncRNA expression participates in carcinogenesis by disrupting major biological processes, such as redirecting chromatin remodeling complexes or inactivating major tumor suppressor genes. We also describe the potential role of dysregulated T-UCRs in cancer, and place these unique RNAs in the lncRNA category. Finally, we note the potential utility of lncRNAs in cancer as diagnostic and prognostic markers, as well as the potential of developing lncRNA mediated therapy.

## Abbreviations

*ANRIL*: Antisense Non-coding RNA in the INK4 Locus; AR: androgen receptor; CREB: cAMP response element binding protein; *DD3*: Differential Display Code 3; HBV: hepatitis B virus; HCC: hepatocellular carcinoma cell lines; *HOTAIR*: HOX Antisense Intergenic RNA; *HULC*: Highly Upregulated in Liver Cancer; IGF2: Insulin-Like Growth Factor 2; kb: kilobase; lincRNA: long or large intergenic non-coding RNA; lncRNA: long non-coding RNA; LTR: long terminal repeat; *MALAT1*: Metastatis-Associated Lung Adenocarcinoma Transcript 1; *MEG3*: Maternally Expressed Gene 3; miRNA: microRNA; ncRNA: non-coding RNA; nt: nucleotide; *PCNCR1*: Prostate Cancer Non-Coding RNA 1; PEI: polyethylenimine; PRC: polycomb repressive complex; *PTEN*: phosphatase and tensin homolog; *PTENP1*: phosphatase and tensin homolog pseudogene 1; RNase: ribonuclease; RNA pol II: RNA polymerase II; RNA pol III: RNA polymerase III; RNAi: RNA interference; SR: serine-arginine rich splicing proteins; TGS: transcriptional gene silencing; tiRNA: transcription initiation RNAs; T-UCR: transcribed ultraconserved region; UTR: untranslated region; *XIST*: X-Inactive-Specific Transcript.

## Competing interests

The authors declare that they have no competing interests.

## Authors' contributions

EAG wrote the first draft of the article, CJB and WLL finalized the manuscript. All authors read and approved the final manuscript.

## Authors' information

EAG is a research fellow at the BC Cancer Agency Research Centre, specializing in non-coding RNA gene structure and function. CJB is a professor of Medical Genetics at the University of British Columbia. Her research focuses on human X chromosome inactivation, in particular the role of *XIST*, a gene she first described during postdoctoral studies in the Willard lab. WLL is a professor of Pathology and Laboratory Medicine at the University of British Columbia. His team invented tiling path array technologies for whole genome and methylome analyses.
